# Alliance Against Cancer, the network of Italian cancer centers bridging research and care

**DOI:** 10.1186/s12967-015-0711-x

**Published:** 2015-11-14

**Authors:** Paolo De Paoli, Gennaro Ciliberto, Manlio Ferrarini, PierGiuseppe Pelicci, Paolo Dellabona, Francesco De Lorenzo, Alberto Mantovani, Pellegrino Musto, Giuseppe Opocher, Piero Picci, Walter Ricciardi, Ruggero De Maria

**Affiliations:** Centro di Riferimento Oncologico (CRO), IRCCS, Istituto Nazionale Tumori, Via Franco Gallini 2, 33081 Aviano, PN Italy; Istituto Nazionale Tumori, Fondazione Pascale, IRCCS, Naples, Italy; Azienda Ospedaliera-Universitaria San Martino-IST Istituto Nazionale per la Ricerca sul Cancro, Genoa, Italy; Istituto Europeo di Oncologia (IEO), Milan, Italy; IRCCS Ospedale San Raffaele, Milan, Italy; Associazione Italiana Malati di Cancro (AIMaC), Rome, Italy; Centro di Riferimento Oncologico della Basilicata (CROB) IRCCS, Rionero in Vulture, PZ Italy; Istituto Oncologico Veneto, IOV IRCCS, Padua, Italy; Humanitas University, Istituto Clinico Humanitas, Rozzano, MI, Italy; Istituto Ortopedico Rizzoli, IRCCS, Bologna, Italy; Istituto Superiore di Sanità, Rome, Italy; Regina Elena National Cancer Institute, Rome, Italy

**Keywords:** Italy, Network of cancer centers, Translational research, Cancer care, Personalized medicine, Clinical trials

## Abstract

Alliance Against Cancer (ACC) was established in Rome in 2002 as a consortium of six Italian comprehensive cancer centers (Founders). The aims of ACC were to promote a network among Italian oncologic institutions in order to develop specific, advanced projects in clinical and translational research. During the following years, many additional full and associate members joined ACC, that presently includes the National Institute of Health, 17 research-oriented hospitals, scientific and patient organizations. Furthermore, in the last three years ACC underwent a reorganization process that redesigned the structure, governance and major activities. The present goal of ACC is to achieve high standards of care across Italy, to implement and harmonize principles of modern personalized and precision medicine, by developing cost effective processes and to provide tailored information to cancer patients. We herein summarize some of the major initiatives that ACC is currently developing to reach its goal, including tumor genetic screening programs, establishment of clinical trial programs for cancer patients treated in Italian cancer centers, facilitate their access to innovative drugs under development, improve quality through an European accreditation process (European Organization of Cancer Institutes), and develop international partnerships. In conclusion, ACC is a growing organization, trying to respond to the need of networking in Italy and may contribute significantly to improve the way we face cancer in Europe.

## Background

Cancer care is a major health problem worldwide. Many countries have recognized that more effective prevention, diagnosis and treatment of cancer require substantial improvements in the coordination of clinical centers and new initiatives to improve our understanding of both clinical and biological aspects of cancer. Prerequisites for success include a sufficient critical mass of infrastructures for cancer care and research, the existence of qualified molecular profiling laboratories and a sound scientific international background and reputation in cancer research. To address these issues, various initiatives are being developed in Europe and/or worldwide. As an example, the basic idea behind Cancer Core Europe, a consortium including six cancer centers from different European Countries (EU), is to build a virtual cancer Institute that allows to increase research coordination, emphasis on the development of personalized oncology to apply state of the art technology to patient care, the creation of an appropriate legal and ethical framework to encourage collaboration between all stakeholders of cancer continuum [1–3]. *The WIN Consortium* represents a further example of global collaboration among cancer centers, life science and biotech organizations, pharmaceutical and technology companies, health plans, and patient advocacy groups coming together from four different continents [4]. This consortium offers an opportunity to make an impact on personalized cancer therapy across the globe by increasing the number of patients, whose malignant cells have been profiled with cutting edge genomic and transcriptomic technologies, to be recruited to innovative clinical trials and treatment with the latest innovative drugs [5]. In this paper, we present the approach that is being undertaken in Italy by ACC to build up a virtual cancer institute spanning across the whole country with the intent to achieve high and uniform standards of cancer research and care, with particular emphasis on personalized cancer therapy based on genomic profiling and on innovative clinical trials.

## Brief history of ACC

**Fig. 1:**
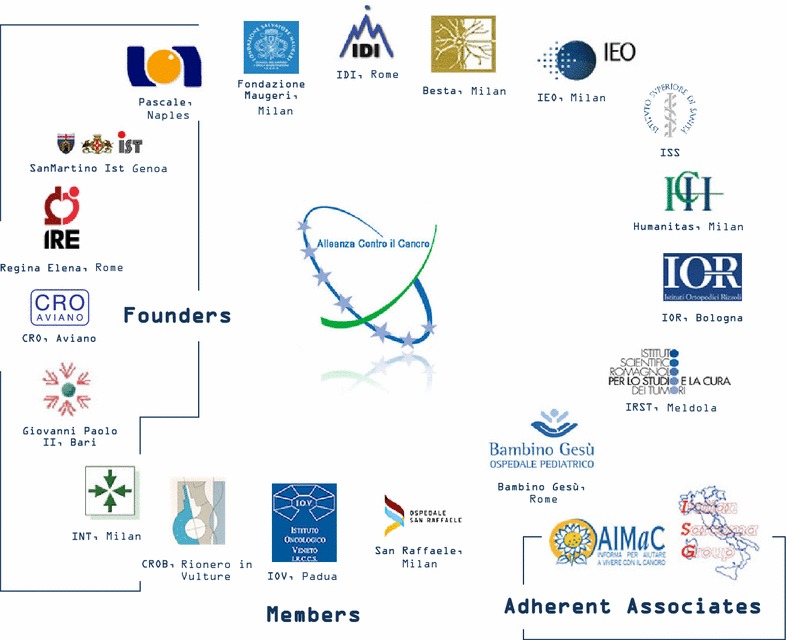
Alliance Against Cancer. The Network of Alliance Against Cancer (ACC) is composed of 20 members, including 6 founders, 10 full members and 2 associate members

ACC was established in Rome in 2002 as a consortium of six Italian comprehensive cancer centers (Founders) recognized by the national Ministry of Health (MoH) as high standard Institutes for both patient care and research (Istituti di Ricovero e Cura a Carattere Scientifico-IRCCS). IRCCS are formally defined by a national law stating that they represent institutions of national relevance pursuing research, mainly clinical and translational, and high quality diagnostic and therapeutic procedures. The aims of ACC were to promote a network among Italian oncologic institutions in order to develop specific, advanced projects in clinical and translational research. With the purpose to generate synergies also with other specialties involved in health care, ACC was chaired by the President of the National Institute of Health. During the first years of activity, ACC developed projects in three areas: a) diagnosis and therapy of cancer b) basic and clinical oncologic research c) education and information in oncology. Within these three areas, some innovative ideas were included, like the constitution of networks in telepathology, rare cancers, quality of life and the molecular classification of solid cancers (genomics and proteomics). During the following years, many additional full and associate members joined ACC, that presently includes 20 members (Fig. 1). A farsighted idea was the inclusion of patients’ associations within the active members and in the assembly of this association, a situation that is now becoming usual in national and international associations or initiatives and scientific societies for cancer care and research [4, 6]. Thanks to the collaboration with cancer patients organizations, ACC has realized the National Cancer Information Service (SION). In its initial configuration, ACC was not organized as an official entity according to national laws, a situation that consistently limited its activities. For this reason, over the last three years ACC underwent a reorganization process that redesigned the structure, governance and major activities. The President is nominated every five years by the Minister of Health among the list of translational scientists selected by the scientific directors of the Founders Institutes, while ACC is now a formal legal entity that can interact with companies and can apply to national and international profit and nonprofit research grants. This is in line with the new major commitment of ACC that concerns the translation of research innovation into clinical trials and routine clinical practice.

The ACC network has now 20 members, including six founders, 12 full and two adherent members (Fig. 1), where approximately a total of 3000 scientists are involved in translational and clinical research, producing publications in peer reviewed international journal for 21,000 Impact Factor Points/year. A distinguishing feature is the high emphasis to clinical trials, with more than 2800 active trials and more than 45,000 patients presently enrolled.

## Qualifying aspects of ACC and current strategy

The most qualifying elements of this alliance, may be considered as follows.

- Within the ACC consortium, all cancer IRCCS officially recognized by the Italian MoH are present independent on their size and geographical location, according to a principle of inclusiveness and equality. This represents the willingness to establish similar standards of care and quality of research across the entire country;
- These centers have recently undergone international European Organization of Cancer Institutes (OECI) accreditation (see below);
- ACC does not include only centers dedicated to cancer, but also other IRCCS in which other medical disciplines (i.e. orthopedics, neurology, dermatology) are present. This approach favors the coverage to a greater extent of rarer types of cancer such as sarcomas, brain tumors, skin cancers etc.;
- The presence within ACC of the Italian Cancer Patients’ Organization (AIMaC) provides a bidirectional information exchange between patients and institutes with a continuous stimulus to look after patient’s needs;
- ACC governance allows sustainability of the consortium because each member is providing basic funding for ACC functioning through its balance.

The main goal of ACC is to achieve high standards of care across the country, implement and harmonize principles of modern personalized and precision medicine, by developing cost effective processes. This will be achieved through a defined set of initiatives of efforts in genomic medicine, new clinical trials, quality of cancer care, and development of international collaborations at the European and worldwide level.

We herein summarize the major initiatives that currently characterize ACC.

1. Tumor genetic screening programs. The first step of this program was the establishment of the ACC Genomics, a group including representatives from different ACC members working together to reach the following goals: a) to establish a shared strategy among all centers for genomic profiling of cancer patients by next generation sequencing of driver and actionable mutations; b) to establish network genomic profiling programs using quality assurance protocols to monitor implementation of new test regulations and to stimulate laboratories to improve their testing procedures; c) to build up a permanent DNA mutation registry of cancer patients treated in the participating centers open for consultation to all members of the Consortium. Through the collection of a high number of DNA profiles, we expect to identify sufficient patients whose cancers have specific genetic alterations to allow their recruitment into personalized medicine clinical trials.
2. ACC has recently established working groups (WG) for each of the six major cancer types for which there is a strong expertise among the network: lung, breast and colorectal cancers, melanoma, glioblastoma and sarcomas. Each WG includes two members from each center, either preclinical or clinician scientists, to facilitate the cancer care-translational research continuum. The final goal of these WG is to establish clinical trial programs for cancer patients treated in Italian cancer centers and to facilitate their access to innovative drugs under development. The existence of the ACC network and shared governance and the existence of common nationally shared regulatory rules overcomes some of the hurdles that limits international studies including countries with different rules/regulations [5].
3. European designation and accreditation of cancer centers. Presently, OECI has developed the most commonly used accreditation program at the European level for cancer centers, with more than 25 institutions having started the Accreditation and Designation procedure (A&D). The OECI A&D program includes standards on general management, screening and prevention, care, research and on patients involvement. Presently all the ACC active members at the moment of application (2012) are part of this program, meaning that Italy is the only European country that has considered essential to share an internationally recognized, high quality, accreditation system for cancer centers [7].
4. Quality approach to colorectal cancer care. Clinical quality can be measured in several manners including with audits by specialized teams on selected types of cancer. Since this approach can be most accurate and reliable, pilot audits have been carried out on the procedures employed in some of the ACC cancer centers. Given its proven feasibility and the interesting data collected, this methodology will be extended to other cancer centers and to other cancer types at the national level [8].
5. Facilitate SION improvement and expansion to meet the increasing demand for information [9].

## International outlook of ACC

ACC is not conceived as a close endeavour including only Italian components, but rather as a large Consortium born to be engaged in International partnerships. Current international activities of ACC can be summarized as follows:

1. Sustaining the European area of translational cancer research. Transcan is an ERANet project aiming at linking translational cancer research funding programs of EU member states and associated countries. Recognizing the strategic importance of this initiative and aiming to improve its internationalization, ACC decided to be part of the Transcan International consortium and became a funding body of Transcan2 by devolving part of its budget to financially support projects starting from the 2014 call on Tumor Heterogeneity [10].
2. ACC has made substantial efforts in the improvement of cancer care at the European level through the participation and financial support to Benchcan, an OECI project co funded by the Health Programme of the European Union [11]. The general objective of Benchcan is to benchmark comprehensive cancer care and yield best practice examples in a way that contributes to improving the quality of interdisciplinary patient treatment. To achieve this, the project addresses six specific objectives:

1. To collect, compare and align by consensus formation the standards, recommendations and accreditation criteria of comprehensive cancer care in selected European countries.
2. To review and refine a benchmarking tool that can be applied to comprehensive cancer care through interdisciplinary patient treatment.
3. To pilot the benchmark tool with particular attention to operations management and best clinical practice.
4. To maximize knowledge exchange and sharing of best practice between providers of comprehensive cancer care in member states and regions.
5. To ensure compatibility of the benchmarking tools with existing cancer care resources and services.
6. To ensure the sustainability and longer-term benefits of the project.

The pilot phase has been already concluded and results are under evaluation and reporting.

3. ACC is entering the MD Anderson Sister Institution Network Program, a global network of major cancer institutions from 23 countries in five continents. This partnership involves translational research, drug development and clinical trials. The impact of such partnership appears of great potential for the development of innovative clinical trials, which may benefit considerably from the availability of a large number of fully characterized patients coupled with the strong expertise of MD Anderson in clinical trials. In this context, the development of common clinical databases may provide a unique opportunity for a strong global clinical partnership.

## Conclusions and future developments

Due to current changes in demographic, social and economic situation, the development of a strategic approach to improve a cancer research and care agenda represents a major challenge for EU countries and worldwide. The new configuration and program of ACC represents a consistent opportunity for Italy. Advantages of ACC are that collaboration is defined by a legal act, is stable, sustained by an annual membership fee of members, supported by MoH and not temporarily linked to projects that have a definite time lapse. At the same time, each partner maintains its operational autonomy and, according to laws, is included in regional healthcare plans, while participating to the development of highly innovative approaches to cancer diagnosis and care. Ultimately, this approach has the potential to greatly increase the number of early clinical trials in Italy and to offer innovative therapies to patients all around the country. Another important effect of ACC strategic plan on genomics, is the possibility to reduce significantly the cost of genomic testing, thus making this an opportunity to be applied widely to all cancer patients in Italy.

Future developments of ACC programs will include the application of genomic approach linked to clinical trials to all other major cancer types, with the aim to provide access to precision medicine clinical trial to metastatic patients unable to be treated successfully with currently approved therapeutic regimens. Furthermore, precision medicine initiatives within ACC will aim to implement integration of genomic platforms with different immunotherapy approaches.

In conclusion, ACC is a growing organization, that is addressing the need of oncology networking in Italy. We foresee that ACC strategy will be a driving force for translational and clinical, while contributing significantly to the internationalization of Italian oncology and the diffusion of high standard clinical practice in Italy.

## Abbreviations

A&D: Accreditation and Designation
ACC: Alliance Against Cancer
AIMaC: Italian Cancer Patients’ Organization
EU: European Union
IRCCS: Institutes for both patient care and research (Istituti di Ricovero e Cura a Carattere Scientifico)
MoH: Ministry of Health
OECI: European Organization of Cancer Institutes
SION: National Cancer Information Service
WG: working groups
